# Vibration reduction of human body biodynamic response in sitting posture under vibration environment by seat backrest support

**DOI:** 10.1038/s41598-024-56109-y

**Published:** 2024-03-18

**Authors:** Wei Ding, Leizhi Wang, Zhaobo Chen, Hongrui Ao, Hui Yan

**Affiliations:** 1 School of Mechatronics Engineering, Harbin Institute of Technology, Harbin, 150001 Heilongjiang People’s Republic of China; 2 Laboratory of Vibration and Noise Control, School of Mechatronics Engineering, Harbin Institute of Technology, Harbin, 150001 Heilongjiang People’s Republic of China

**Keywords:** Backrest, Human–chair coupling model, STHT, Mechanical engineering, Applied mathematics

## Abstract

Four-degree-of-freedom (4-DOF) human–chair coupling models are constructed to characterize the different contact modes between the head, chest back, waist back and backrest. The seat-to-head transfer ratio (STHT) is used as an evaluation metric for vibration reduction effectiveness. The simulated vibration reduction ratio of the model is close to the experimental results, which proves the validity of the model. The peak STHT is obviously reduced (P < 0.05, T-test) with seat-backrest support. The experiments show that supporting the head (${a}_{1}$, P < 0.05, Wilcoxon matched-pairs signed ranks) has the best vibration reduction effect (21%), supporting the chest back (${a}_{2}$, P < 0.05) has a reduced effect (11%), and supporting the waist back (${a}_{3}$, P < 0.05) has the weakest effect (4%). When the upper torso is in full contact with the backrest, the peak STHT curve and resonance frequency are positively correlated with the contact stiffness of the seat surface and negatively correlated with the contact damping. In order to reduce the seat-to-head transfer ratio, the lowest STHT peak and lowest total energy judgments were proposed as the selection methods for the selection of the contact stiffness and damping of the backrest in two environments (periodic and non-periodic excitation), respectively.

## Introduction

Long-term human exposure to vibration can lead to lumbar erector spinae muscle fatigue, lumbar muscle damage, changes in the physiological structure of the lumbar spine, and finally, a variety of chronic diseases that cause permanent damage to the torso^1–4^. Countries around the world have consumed significant medical resources in this area. Kelsey’s study^5^ made a direct link between the human biodynamic response in vibrating environments and human health status.

The most intuitive way to mitigate the response of the seated human body in a vibration environment is through the design of a proper seat. Currently, some transportation seats have high vibration intensities, such as helicopters up to 0.1 to 0.2 g^6^, but transportation seats cannot effectively reduce vibration. Many seats use modular plastic seats to reduce costs, which have neither vibration reduction nor headrests. Some seats are covered with cotton padding, but the backrests are significantly protruding, which is not conducive to the chest back and waist back resting against the backrest at the same time. A few premium seats have a backrest design that hugs the curve of the spine, but the headrest protrudes too far forward for the occupant’s usual comfort. Most transportation seats, in short, have problems with uneven upper torso support because the backrest does not conform to the body’s curves.

**Figure 1:**
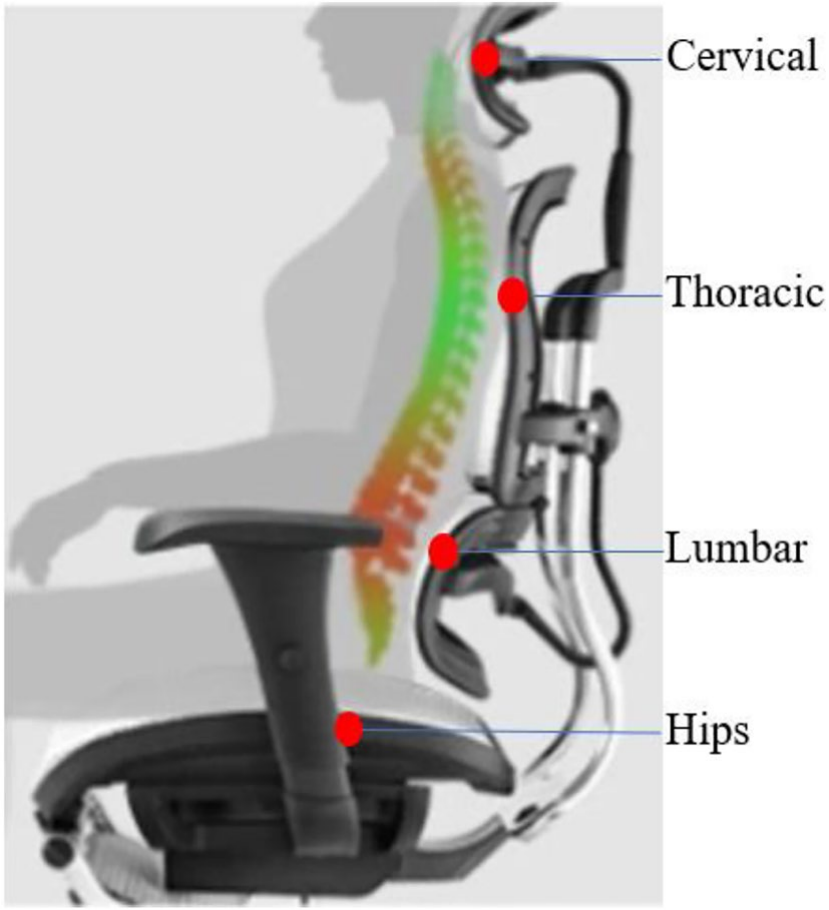
Effective support point position of the upper torso by the ergonomic chair.

In recent years, for indoor sedentary people, the market launched an ergonomic chair to maintain a healthy physiological curvature of the spine, to alleviate the fatigue caused by sedentary activity. One of the characteristics of ergonomic chairs that is different from ordinary chairs is that its backrest is designed along the curve of the human spine, while the headrest is adjustable, so it can provide effective support for the human head, chest back, and waist back from the cervical, thoracic, and lumbar vertebral points (Fig. 1). Such a seat backrest design can support the human body in a balanced way, so it will relieve fatigue more than ordinary seats. It is worth exploring whether providing effective support to the head, chest back and waist back of the seated human body in a vibrating environment can provide good vibration reduction effect.

Modelling the human body to predict human biodynamic responses is an important tool for studying human health and comfort. The main postures studied in human biodynamics are the standing posture^7^ and the sitting posture^8^. In this paper, we study the effect of the backrest on the vibration reduction of a seated human body using the upper torso model.

The lumped parameter model aims to predict the vibration and impact dynamics response of a seated human body by dividing the body into mass blocks connected by stiffness and damping. It has the advantage of integrating excitation sources and is therefore suitable for the development of vibration reduction in seats^9^ and for the prevention of impact injuries^10^. In the study of modelling methods, researchers have proposed refinement directions such as the number of degrees of freedom, with different degrees of freedom such as 1^11^, 2^12^, 4^13^, 7^14^, 11^15^, linear versus nonlinear^16^, the internal force of human respiration^14^, and the tilt angle of the human body^17^.

For the seat excitation, most experiments had the excitation touching only the hips. In fact, the presence of a backrest not only leads to a change in the state of sitting, but additionally provides incentives to the body. It has been experimentally demonstrated that the sitting state and hand and foot positions^18–20^ cause changes in the resonance frequency. The body relaxes when leaning on the backrest compared to sitting without a backrest, and this leads to speculation that the vibration response is related to the body state. This was confirmed by a study by Adam^21^.

Multiple model structures explain the diversity of biodynamic responses of the human body. The human body is able to maintain a homeostatic response when the occupant maintains a single seated position and keeps the muscle state constant. However, real people are not able to do this, let alone subjects who are emphasized to be unsupported by a backrest. Over time, the vibrating seat surface gradually changes the body’s sitting posture, resulting in altered muscle states and fatigue. Backrests reduce the burden on the human torso. In other researchers’ vibration experiments, the backrest factor was either excluded or its role was ignored. However, life experiences with transportation can demonstrate that vibrational discomfort is reduced when the body leans backrest. The vibration reduction effect of the seat backrest cannot be ignored.

No studies have yet examined the role of seat backrest in vibration reduction. What kind of role the backrest plays in the vibration environment for the human biodynamic response needs to be studied in depth. Using the human–chair coupling model to study the vibration reduction effect of the seated human body in contact with the backrest, and the stiffness and damping of the backrest on the vibration reduction effect of the influence is the focus of this paper.

To investigate the vibration reduction effect of the backrest, 4-DOF models are developed to characterize the backrest support to the head, chest back and waist back of the seated human body. The response curves of different sitting postures are calculated through simulation. Vibration experiments are carried out for the four sitting postures in which the muscles easily maintain the same state, and the validity of the model is verified by comparing the results of theoretical calculations and experiments. The contact stiffness and damping of the backrest are changed to calculate the vibration response of the full-contact model, and the effects of these two parameters on the seat-to-head transfer (STHT) ratio are investigated. According to whether the excitation is periodic or nonperiodic, the selection methods of the contact stiffness and damping of the backrest are given to effectively reduce the vibration response and improve the comfort.

## 4-DOF human biodynamic models

**Figure 2:**
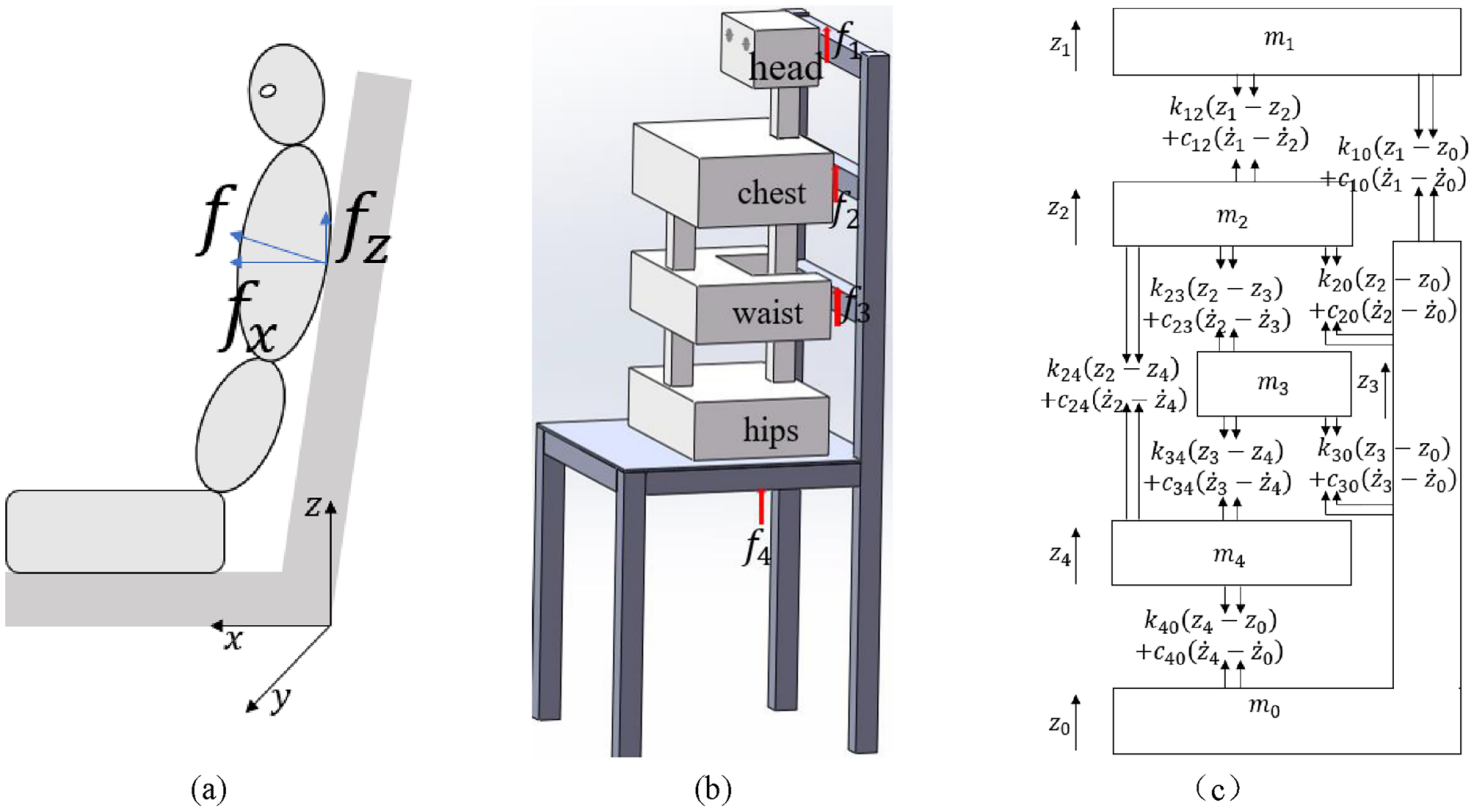
4-DOF full-contact backrest human-chair coupling model.

Seat backrests usually have a small inclination angle and normal support to the human body, as shown in Fig. 2a. In this paper, we study the vibration response in the vertical direction, so we neglect its lateral component force. According to the four support points in Fig. 1, the full contact backrest is modelled as shown in Fig. 2b. The 4-DOF lumped parameter model is shown in Fig. 2c, with ${m}_{0}$–${m}_{4}$ representing the seat, head, chest, waist and hips, respectively. To simplify the study, the vertical constraints of the backrest are replaced with forces due to stiffness ${k}_{i0}$ and damping ${c}_{i0}$ (*i* = 1, 2, 3). Since the chair surface materials are the same, ${k}_{10}={k}_{20}={k}_{30}={k}_{40}$, ${c}_{10}={c}_{20}={c}_{30}={c}_{40}$. When the head, chest back and waist back are not supported, this is the model of Wan^13^, and its parameters are shown in Table 1.

**Table 1:** 4-DOF human biodynamic model parameters^13^.

Mass/(kg)				Stiffness/(10^3^ N/m)					Damping/(10^2^ N s/m)				
${m}_{1}$	${m}_{2}$	${m}_{3}$	${m}_{4}$	${k}_{12}$	${k}_{23}$	${k}_{34}$	${k}_{40}$	${k}_{24}$	${c}_{12}$	${c}_{23}$	${c}_{34}$	${c}_{40}$	${c}_{24}$
4.170	15.00	5.500	36.00	134.4	10.00	20.00	49.34	192.0	2.500	2.00	3.300	24.75	9.091

The human body and the backrest are not necessarily in complete contact, so different sitting postures can be modelled from Fig. 2c by deleting the contact points (${k}_{i0}$ and ${c}_{i0}$). There are eight specific sitting postures:

- ${a}_{0}$: no contact between upper torso and seat backrest;
- ${a}_{1}$: head in contact with the seat backrest (The model contains ${k}_{10}$, ${c}_{10}$);
- ${a}_{2}$: chest back in contact with the seat backrest (The model contains ${k}_{20}$, ${c}_{20}$);
- ${a}_{3}$: waist back in contact with the seat backrest (The model contains ${k}_{30}$, ${c}_{30}$);
- ${a}_{4}$: head and chest back in contact with the seat backrest (The model contains ${k}_{10},{c}_{10},{k}_{20},{c}_{20}$);
- ${a}_{5}$: head and waist back in contact with the seat backrest (The model contains ${k}_{10},{c}_{10},{k}_{30},{c}_{30}$);
- ${a}_{6}$: chest back and waist back in contact with the seat backrest (The model contains ${k}_{20},{c}_{20},{k}_{30},{c}_{30}$);
- ${a}_{7}$: head, chest back and waist back in contact with the seat backrest (The model contains ${k}_{10}$, ${c}_{10}$, ${k}_{20}$, ${c}_{20}$, ${k}_{30}$, ${c}_{30}$).

The force analysis of each mass block of the full contact model is shown in Fig. 2c. According to Newton’s second law, their equation of motion can be expressed as Eq. (1):

1 $$\left\{ \begin{aligned} m_{1} \ddot{z}_{1} + & \;k_{12} \left( {z_{1} - z_{2} } \right) + c_{12} \left( {\dot{z}_{1} - \dot{z}_{2} } \right) + k_{10} \left( {z_{1} - z_{0} } \right) + c_{10} \left( {\dot{z}_{1} - \dot{z}_{0} } \right) = 0 \\ m_{2} \ddot{z}_{2} - & \;k_{12} \left( {z_{1} - z_{2} } \right) - c_{12} \left( {\dot{z}_{1} - \dot{z}_{2} } \right) + k_{23} \left( {z_{2} - z_{3} } \right) + c_{23} \left( {\dot{z}_{2} - \dot{z}_{3} } \right) + k_{24} \left( {z_{2} - z_{4} } \right) \\ + & \;c_{24} \left( {\dot{z}_{2} - \dot{z}_{4} } \right) + k_{20} \left( {z_{2} - z_{0} } \right) + c_{20} \left( {\dot{z}_{2} - \dot{z}_{0} } \right) = 0 \\ m_{3} \ddot{z}_{3} - & \;k_{23} \left( {z_{2} - z_{3} } \right) - c_{23} \left( {\dot{z}_{2} - \dot{z}_{3} } \right) + k_{34} \left( {z_{3} - z_{4} } \right) + c_{34} \left( {\dot{z}_{3} - \dot{z}_{4} } \right) + k_{30} \left( {z_{3} - z_{0} } \right) \\ + & \;c_{30} \left( {\dot{z}_{3} - \dot{z}_{0} } \right) = 0 \\ m_{4} \ddot{z}_{4} - & \;k_{34} \left( {z_{3} - z_{4} } \right) - c_{34} \left( {\dot{z}_{3} - \dot{z}_{4} } \right) + k_{40} \left( {z_{4} - z_{0} } \right) + c_{40} \left( {\dot{z}_{4} - \dot{z}_{0} } \right) = 0. \\ \end{aligned} \right.$$

Equation (1) is summarized in matrix form:

2 $${[M]}_{4\times 4}{\left\{\ddot{z}\right\}}_{4\times 1}+{\left[C\right]}_{4\times 4}{\left\{\dot{z}\right\}}_{4\times 1}+{\left[K\right]}_{4\times 4}{\left\{z\right\}}_{4\times 1}={\left\{{f}_{z}\right\}}_{4\times 1},$$

where $\left[M\right]$, $\left[C\right]$ and $\left[K\right]$ are the 4 × 4 mass, damping and stiffness matrices, respectively; $\ddot{z}$, $\dot{z}$ and $z$ are the acceleration, velocity and displacement vectors of the mass block; and ${f}_{z}$ is the force vector generated by seat displacement.

$${M}_{{a}_{7}}=\left[\begin{array}{cccc}{m}_{1}& 0& 0& 0\\ 0& {m}_{2}& 0& 0\\ 0& 0& {m}_{3}& 0\\ 0& 0& 0& {m}_{4}\end{array}\right],$$

$${K}_{{a}_{7}}=\left[\begin{array}{cccc}{k}_{12}+{k}_{10}& -{k}_{12}& 0& 0\\ -{k}_{12}& {k}_{12}+{k}_{23}+{k}_{24}+{k}_{20}& -{k}_{23}& -{k}_{24}\\ 0& -{k}_{23}& {k}_{23}+{k}_{34}+{k}_{30}& -{k}_{34}\\ 0& -{k}_{24}& -{k}_{34}& {k}_{24}+{k}_{34}{+k}_{40}\end{array}\right],$$

$${C}_{{a}_{7}}=\left[\begin{array}{cccc}{c}_{12}+{c}_{10}& -{c}_{12}& 0& 0\\ -{c}_{12}& {c}_{12}+{c}_{23}+{c}_{24}+{c}_{20}& -{c}_{23}& -{c}_{24}\\ 0& -{c}_{23}& {c}_{23}+{c}_{34}+{c}_{30}& -{c}_{34}\\ 0& -{c}_{24}& -{c}_{34}& {c}_{24}+{c}_{34}{+c}_{40}\end{array}\right],$$

$${{f}_{z}}_{{a}_{7}}=\left[\begin{array}{c}{{k}_{10}z}_{0}+{c}_{10}{\dot{z}}_{0}\\ {{k}_{20}z}_{0}+{c}_{20}{\dot{z}}_{0}\\ {{k}_{30}z}_{0}+{c}_{30}{\dot{z}}_{0}\\ {{k}_{40}z}_{0}+{c}_{40}{\dot{z}}_{0}\end{array}\right].$$

The rest of the models differ in that the stiffness and damping at the uncontacted point is 0:

- $${a}_{0}: {k}_{10}=0,{c}_{10}=0,{k}_{20}=0,{c}_{20}=0,{k}_{30}=0,{c}_{30}=0;$$
- $${a}_{1}: {k}_{20}=0,{c}_{20}=0,{k}_{30}=0,{c}_{30}=0$$;
- $${a}_{2}: {k}_{10}=0,{c}_{10}=0,{k}_{30}=0,{c}_{30}=0;$$
- $${a}_{3}: {k}_{10}=0,{c}_{10}=0,{k}_{20}=0,{c}_{20}=0;$$
- $${a}_{4}: {k}_{30}=0,{c}_{30}=0;$$
- $${a}_{5}: {k}_{20}=0,{c}_{20}=0;$$
- $${a}_{6}: {k}_{10}=0,{c}_{10}=0.$$

Equation (2) is solved using the frequency domain method, starting with the Laplace transform:

3 $${S}^{2}\left[M\right]\left\{Z(s)\right\}+S\left[C\right]\left\{Z(s)\right\}+\left[K\right]\left\{Z(s)\right\}=\left[{\text{B}}\right]{\left[\begin{array}{c}1\\ s\end{array}\right]Z}_{0}\left(s\right),$$

where $$B=\left[\begin{array}{cc}{k}_{10}& {c}_{10}\\ {k}_{20}& {c}_{20}\\ {k}_{30}& {c}_{30}\\ {k}_{40}& {c}_{40}\end{array}\right]$$.

Let $[{\text{A}}]={S}^{2}\left[M\right]+S\left[C\right]+\left[K\right]$; then, the transfer rate from the seat to each mass block is as follows:

4 $$H\left(s\right)=\frac{Z(s)}{{Z}_{0}(s)}={[A]}^{-1}\left[{\text{B}}\right]\left[\begin{array}{c}1\\ s\end{array}\right].$$

Let $s=j\omega$; then, the expression for STHT is:

5 $$STHT=\frac{{z}_{1}(j\omega )}{{z}_{0}(j\omega )}.$$

## Effect of backrest support points

In this section, the STHT curves of eight models subjected to vertical excitation are simulated. Experiments are carried out on four sitting postures that are easy to control muscle states. The simulation results and experimental phenomena are discussed.

### Simulation

**Figure 3:**
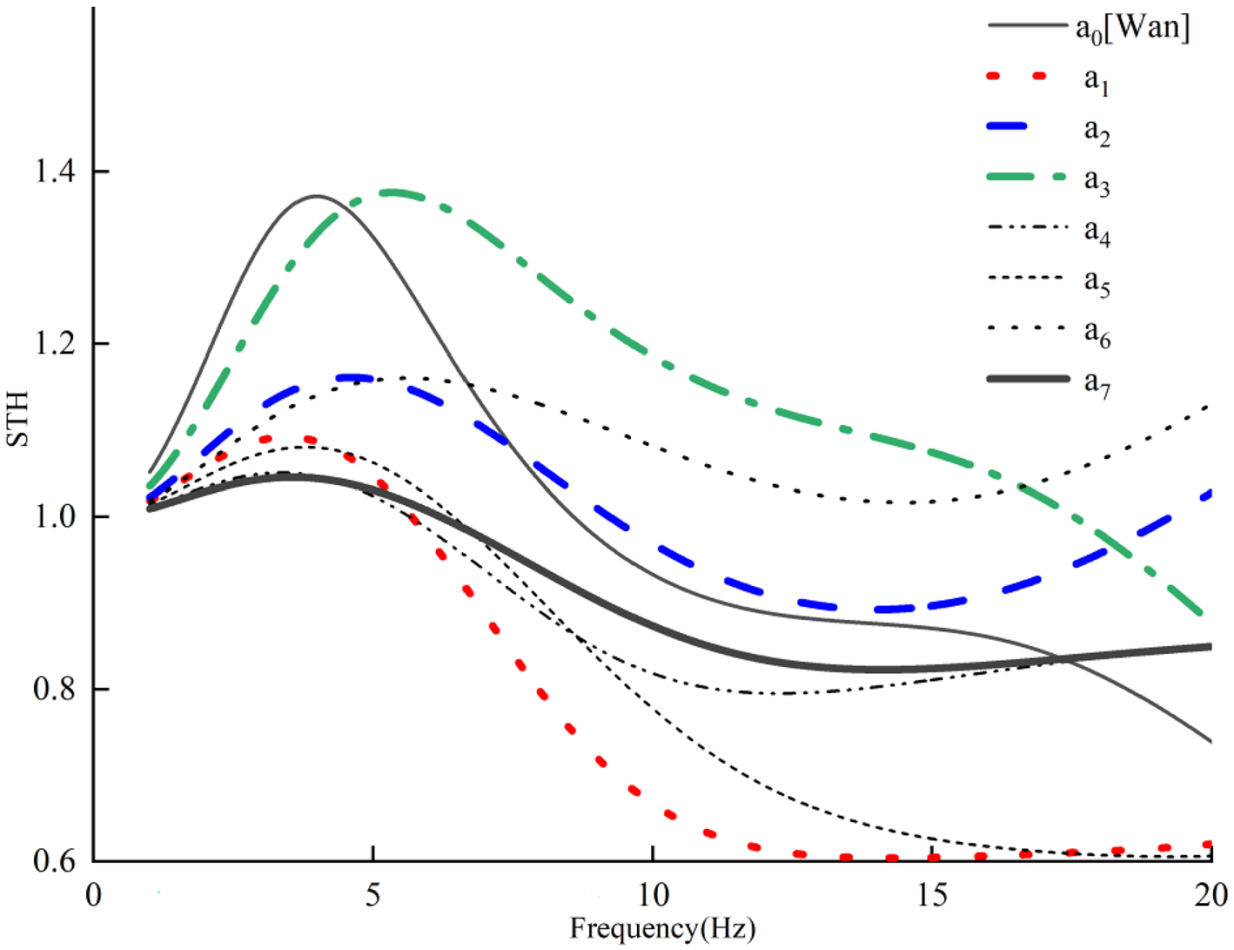
STHT simulation curves for different sitting models.

**Figure 4:**
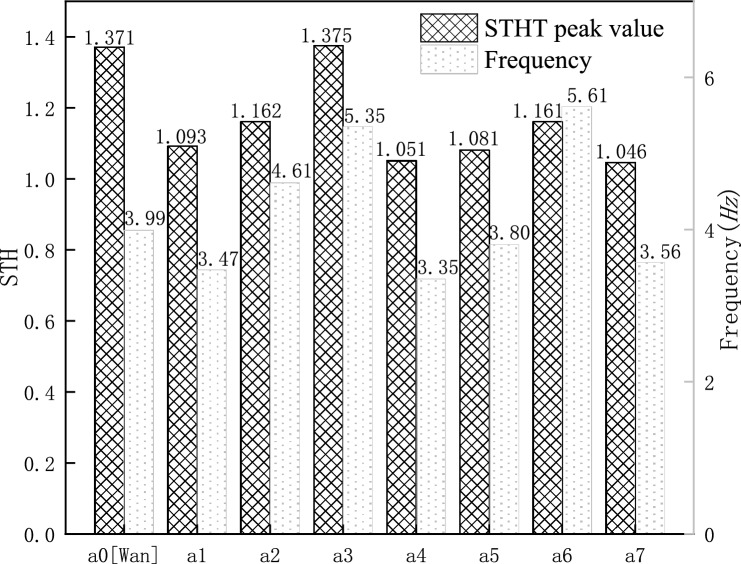
STHT peak values and the resonance frequencies.

The STHT curves of the eight models are shown in Fig. 3 by simulation with displacement excitation applied to the seat from 1 to 20 Hz. The peak STHT values and their resonance frequencies are displayed in Fig. 4.

Figure 3 shows that the morphology, peak and resonance frequencies of the STHT curves changed with backrest support compared to without backrest support (${a}_{0}$ [Wan]). When the waist back and backrest were in contact alone (${a}_{3}$), a second peak in STHT was present and disappeared for the rest of the models. It is therefore reasonable to hypothesize that the second peak of STHT in the experiments without backrest support should be related to the human head and chest back.

Figure 4 shows the specific values of the peak STHT curves and resonance frequencies for each model. Except for model ${a}_{3}$, the peak STHT values of all the models decreased to different degrees. The change in resonance frequency indicates that head contact with the backrest causes the resonance frequency to decrease, and chest back and waist back contact with the backrest causes the resonance frequency to increase.

Table 2 shows the vibration reduction effect (peak value reduction percentage) of the models with backrests. In terms of the vibration reduction effect, the full contact model (${a}_{7}$) is the best, with a peak reduction of 24%. In terms of support sites, supporting the head is the most effective, with all three models (${a}_{1}$, ${a}_{4}$, ${a}_{7}$) showing more than 20% vibration reduction, supporting the chest back is second, and supporting the waist back is the worst.

**Table 2:** Simulated vibration reduction effect of the models with backrest.

Sitting posture	${a}_{1}$	${a}_{2}$	${a}_{3}$	${a}_{4}$	${a}_{5}$	${a}_{6}$	${a}_{7}$
Peak reduction ratio (%)	20	15	0	23	21	15	24

The human body in the lumped parameter model is a structure of multimass lumps connected in series and parallel. The greater the number of constraint points to which the system is subjected, the more effective the constraint is. When multiple points are constrained, the effect of simultaneous constraints at both ends is higher than that in the case where free ends exist.

### Experiment

#### Subjects and experimental equipment

Informed consent was obtained from eight healthy male student volunteers with no medical history who were aware of the study purpose, risks and benefits and signed an informed consent form prior to the experiment; the experiment was approved by the Medical Ethics Committee of Harbin Institute of Technology (Ethics No. HIT-2023032). The research was conducted in accordance with the Declaration of Helsinki. All research respects individual autonomy and uphold the principle of safety without causing harm to subjects. The subject who provided photographs consented to the publication of information and images about him in an online open access publication.

**Figure 5:**
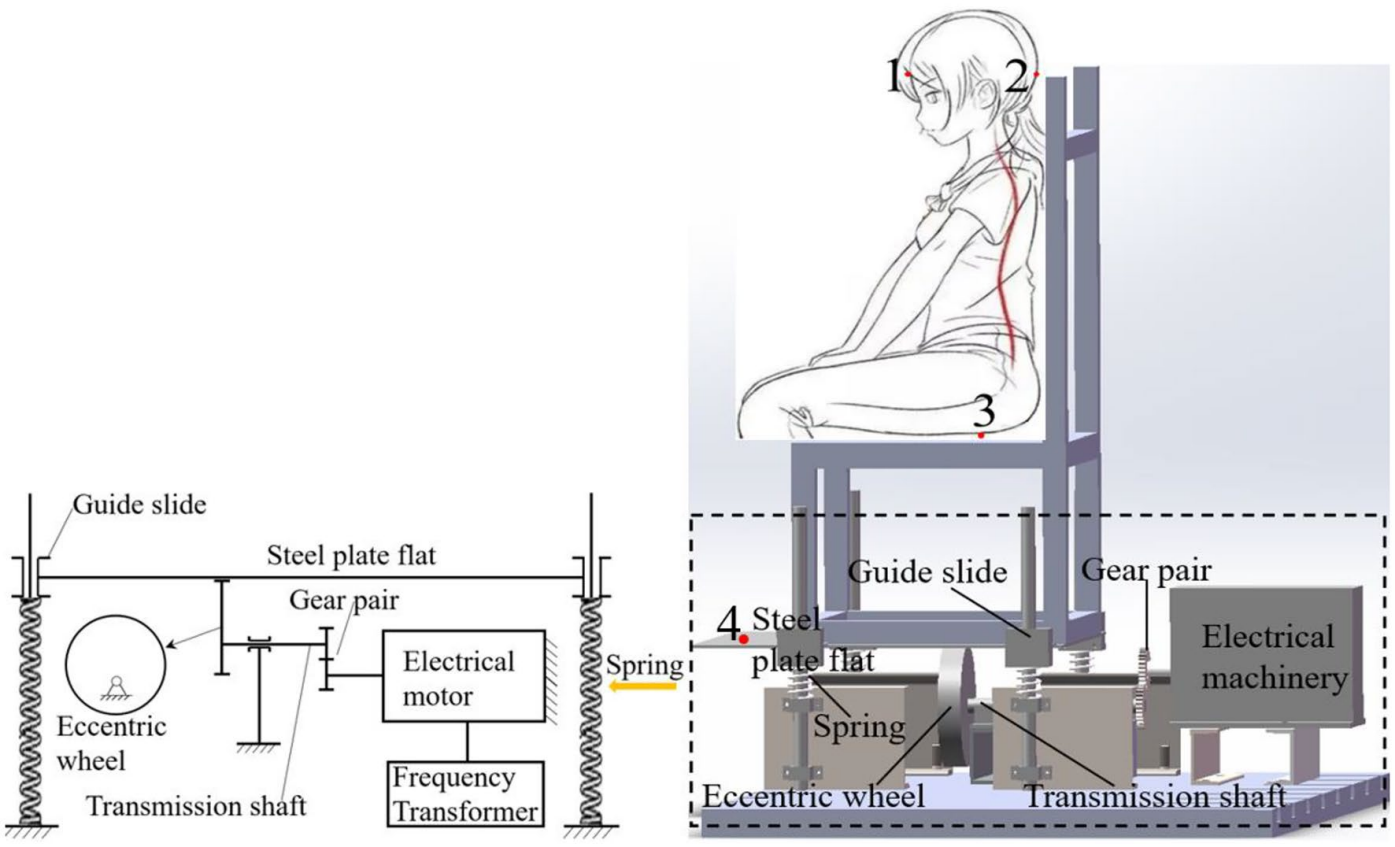
Experimental setup and sensor location.

The physical characteristics of the subjects are summarized in Table 3. The low frequency vertical vibration table for performing the seated human vibration test is shown in Fig. 5. The seat was made of aluminum profiles, and the contact damping between the human body and the seat relied almost entirely on the human body itself. The subjects wore a safety belt to prevent flopping.

**Table 3:** Eight subjects’ physical characteristics.

	Minimum	Maximum	Mean	Standard deviation
Age (years)	25	31	28.25	2.31
Weight (kg)	56.5	95	78.76	12.42
Height (cm)	172	185	178.25	4.71

**Figure 6:**
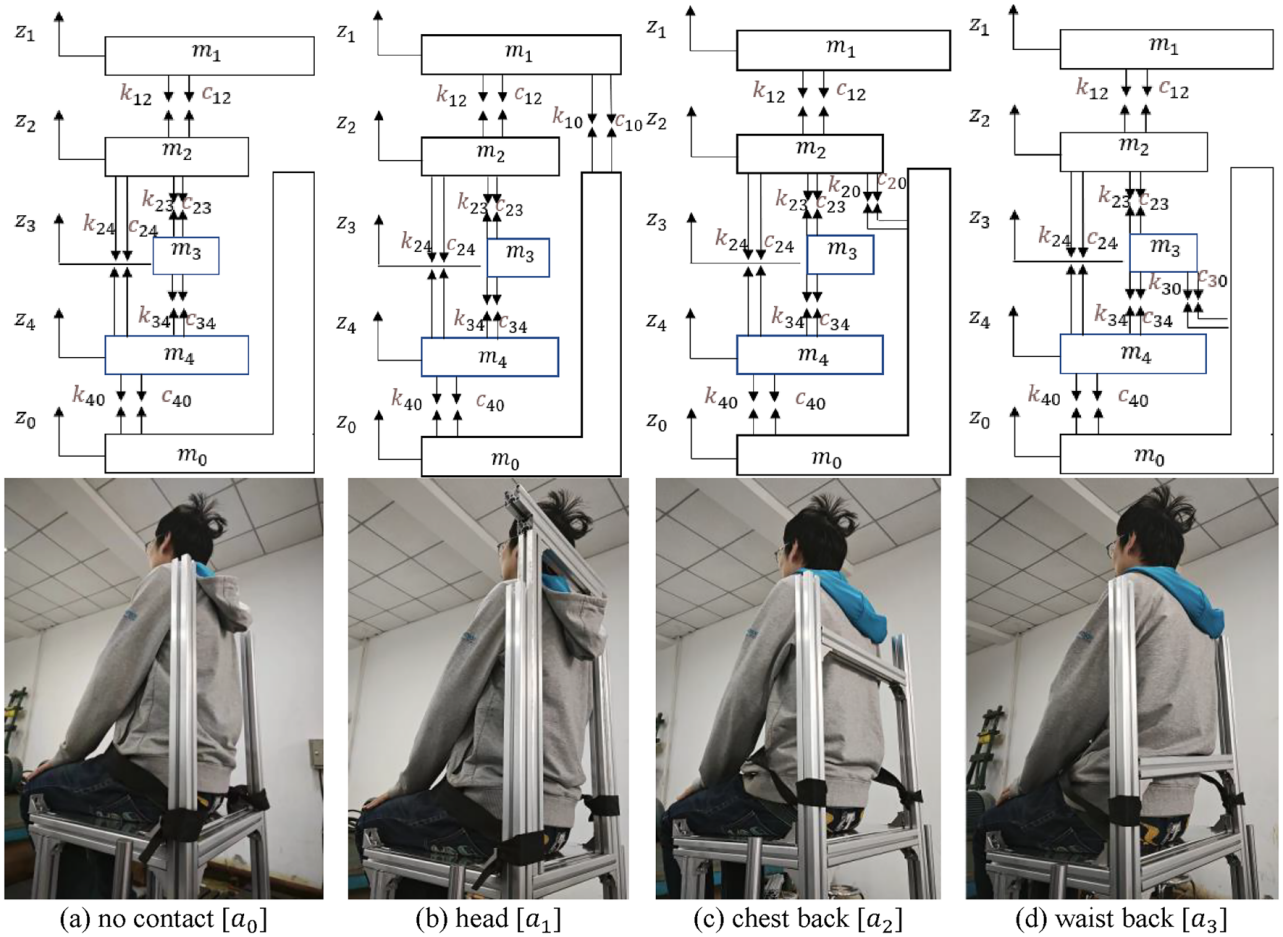
Seated human body in contact with the back of the chair.

Four simple sitting postures that could keep the muscle state stable were adopted for the experiment, as shown in Fig. 6: (a) no contact (${a}_{0}$); (b) support head (${a}_{1}$); (c) support chest back (${a}_{2}$); (d) and support waist back (${a}_{3}$). The relaxed state in the ${a}_{0}$ posture is the reference state that the subjects’ muscles need to maintain as much as possible during the experiment.

The eccentric wheel applies excitation to the steel plat flat, which is a sinusoidal wave with an amplitude A = 3 mm.

6 $${z}_{0}=A{\text{sin}}\left(2\pi ft\right).$$

The acceleration of the excitation is expressed as:

7 $${\ddot{z}}_{0}=-(2\pi f{)}^{2}A{\text{sin}}\left(2\pi ft\right).$$

Among transportation systems, helicopters have large vibrations in normal operation, which can reach 0.1 to 0.2 g^6^. According to Eq. (7), the amplitude of the excitation acceleration applied by the eccentric wheel varies with the frequency *f*. The range of excitation frequency f is set from 1 to 5 Hz, and the excitation signal was set every 0.5 Hz.

Acceleration acquisition was performed using patch sensors (WT9011G4K; Shuyang Zhebaoshennyi E-commerce Co., Ltd., Suqian City, Jiangsu Province, China) with a resolution of 0.0005 g. The data recording points were the fore-head (point 1), the back-head (point 2), the hips in contact with the seat surface (point 3), and the surface of the steel plate (point 4), see Fig. 5. The two sensors on the head were bound with medical bandages. The remaining two sensors were glued to the seat surface and steel plate respectively. In the experiment, the time-domain signals measured at points 3 and 4 were highly similar, and the peaks of the frequency-domain signals were in line with the output frequency of the motor. The seat remains rigidly connected to the steel plate. The chair vibration frequency is the same as the output frequency of the motor.

The sampling frequency was 1.6 kHz. At each sampling point, the ratio of the maximum acceleration of the head to the seat surface was taken as the seat-to-head transfer ratio at that point. Multiple experiments were performed for each sitting posture separately and the results were averaged.

Table 4 shows the vibration duration (s) for each subject at 9 freq. × 4 sitting postures. For each sitting posture, vibration experiments were conducted at 0.5 Hz intervals starting from 1 Hz. The maximum excitation frequency was 5 Hz. Each vibration required the subject to hold the desired position for 45 s to achieve a steady state. Three experiments were conducted for each posture. A 1-min interval between each vibration test was provided for the subject to adjust his/her state.

**Table 4:** Subjects’ vibration duration (s) at 9 freq. × 4 sitting postures.

Sitting posture	Freq. (Hz)								
	1.0	1.5	2.0	2.5	3.0	3.5	4.0	4.5	5.0
${a}_{0}$	45	45	45	45	45	45	45	45	45
${a}_{1}$	45	45	45	45	45	45	45	45	45
${a}_{2}$	45	45	45	45	45	45	45	45	45
${a}_{3}$	45	45	45	45	45	45	45	45	45

#### Results

**Figure 7:**
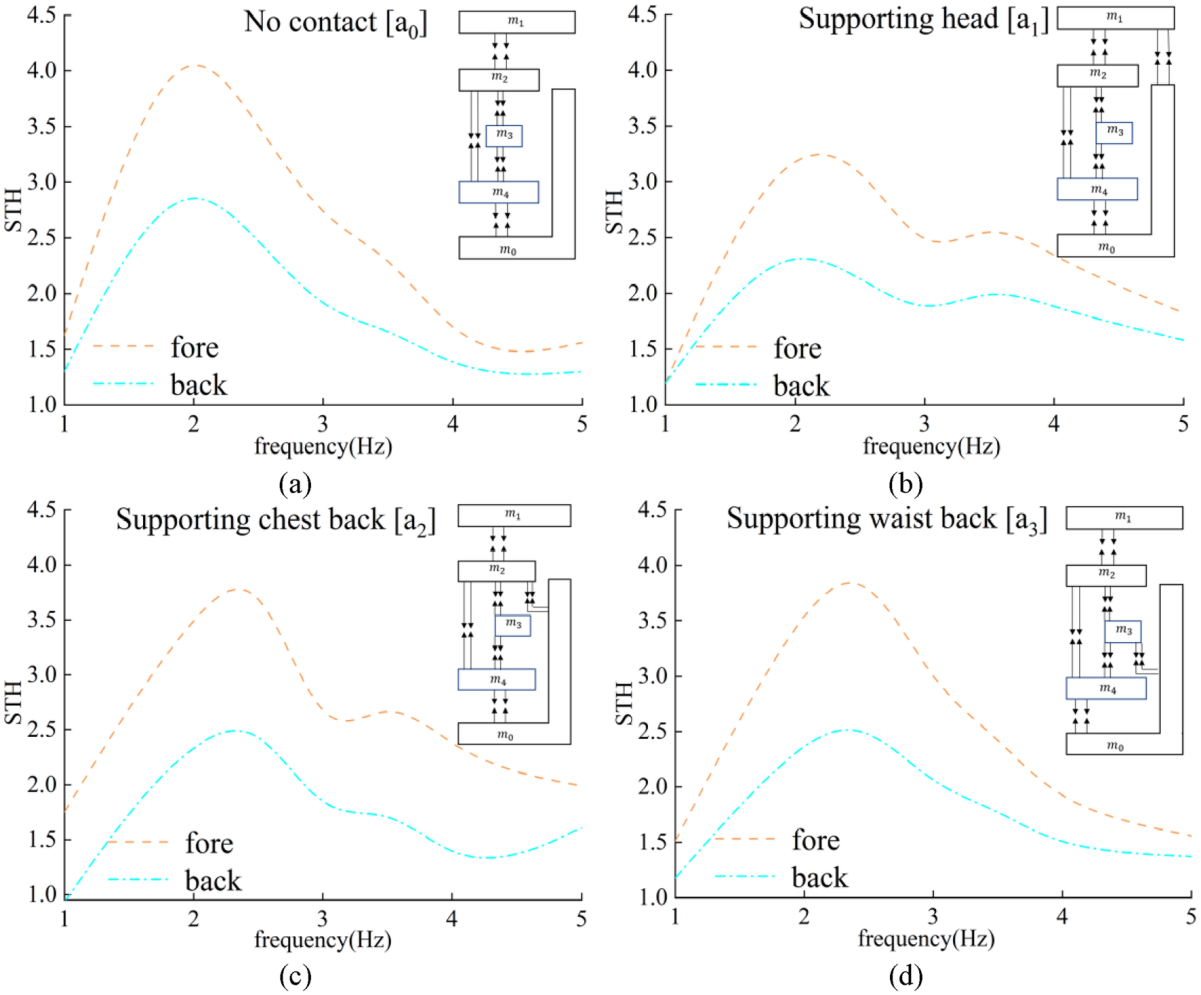
STHT curves of the fore-head and back-head for the 4 sitting postures.

The STHT curves of the fore-head and back-head for each sitting posture are shown in Fig. 7. Averaging the vibration responses of the fore-head and back-head gives the response at the center of mass position of the head. The STHT curves are summarized in Fig. 9. Table 5 shows the Pearson correlations of STHT responses for the four sitting postures. The experimentally measured vibration reduction effects of the model with backrest are shown in Table 6.

**Table 5:** STHT correlation coefficients between the four sitting postures.

	${a}_{0}$	${a}_{1}$	${a}_{2}$	${a}_{3}$
${a}_{0}$		0.893**	0.788*	0.894**
${a}_{1}$			0.881**	0.798*
${a}_{2}$				0.795*

*P < 0.05; **P < 0.01 (two-tailed).

**Table 6:** Comparison of vibration reduction effect between experimentation and simulation.

Sitting posture	${a}_{0}$	${a}_{1}$	${a}_{2}$	${a}_{3}$
Experimental peak	3.45	2.74	3.06	3.30
Experimental peak reduction ratio (%)	–	21	11	4
Simulation peak	1.37	1.09	1.16	1.37
Error in experiments and simulations	–	1	− 4	4

In Fig. 7, at the same frequency, the response curves for the human fore-head are generally higher than those for the back-head, regardless of where the backrest is supported. The STHT curves of the model without backrest support (${a}_{0}$) and the supporting waist back model (${a}_{3}$) are both one peak. The STHT curves of the supporting head model (${a}_{1}$) and the supporting chest back model (${a}_{2}$) both showed two peaks. Compared with the ${a}_{0}$ curve in Fig. 3, where external constraints were added to the head and chest in the experiment, the STHT curve produced a second peak within 5 Hz. This indicates that the frequency of the second peak of the STHT curve is related to the restraint status of the head and chest.

**Figure 8:**
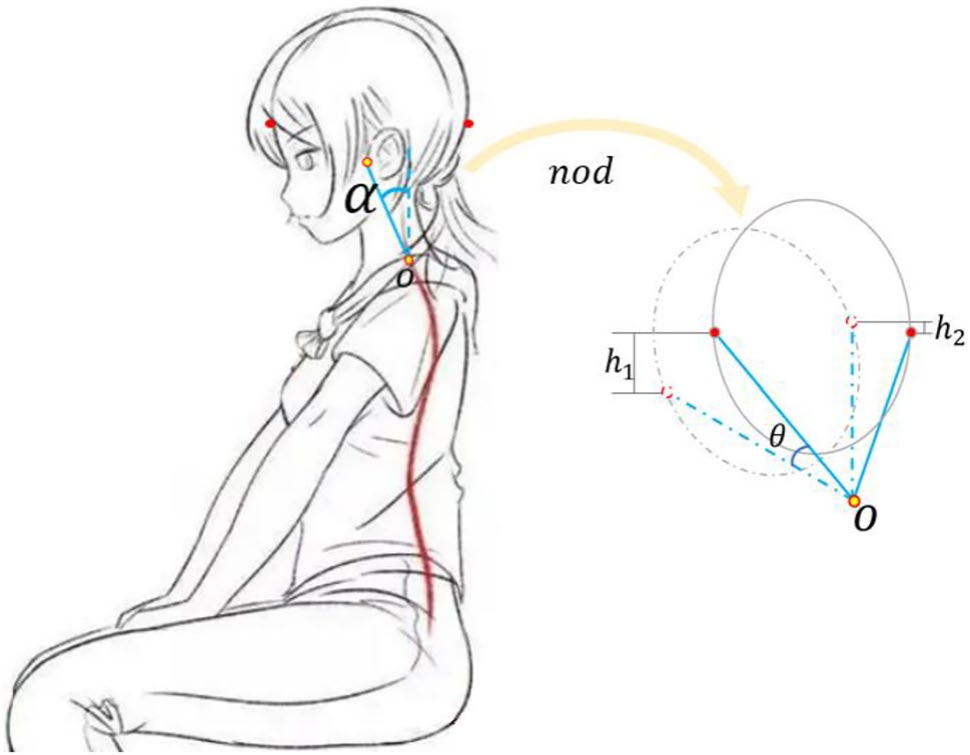
Analysis of the effect of nodding on STHT differences.

In Fig. 7, the fore-head curve is higher than the back-head, which is analysed as a result of nodding. When sitting upright, the horizontal position of the cranial center of mass is roughly on the line between the auricle and the eye. This posture creates a deviation of approximately 2 to 4 cm between the cranial center of mass and the cervical spine support point (Fig. 8), i.e., there is an anterior tilt angle α. Therefore, in the vibration state, in addition to the linear vibration in the direction of the spine, there is a “nodding” oscillation of the head. Decomposing the “nodding” oscillation, there are more vertical components of motion in the front of the head than in the back, and therefore the vertical transmission rate is higher in the frontal.

**Figure 9:**
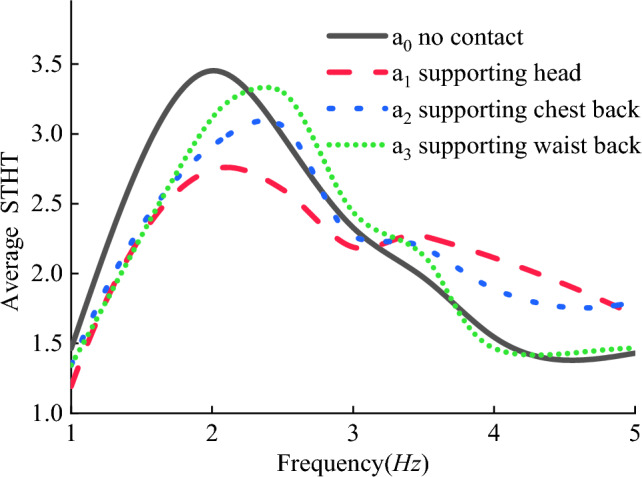
STHT experimental curves for sitting with backrest contact.

As shown in Table 5, there is a significant correlation (P < 0.05) of vibration response between different sitting postures. Figure 9 shows the average values of the STHT of the head in the experiment, with a peak STHT of 3.452 and a resonance frequency of 2.0 Hz for the model without backrest support (${a}_{0}$). When backrest support was present, the peak STHT of the system generally decreased while the resonance frequency changed. In the order that the head, chest back and waist back were supported (${a}_{1}$, ${a}_{2}$, ${a}_{3}$), respectively, the peak values and resonant frequencies increased. This is consistent with the simulation results. The degree of reduction of the peaks in Table 6 shows that supporting the head (${a}_{1}$, P < 0.05, Wilcoxon matched-pairs signed ranks, two-tailed) has the best effect with a 21% reduction, supporting the chest back (${a}_{2}$, P < 0.05) has a reduced effect with an 11% reduction, and supporting the waist back (${a}_{3}$, P < 0.05) has the weakest effect with a mere 4% reduction. The vibration reduction effect of the backrest support is significant. The corresponding reductions in the simulation group were 20%, 15% and 0%, respectively. For the vibration reduction effects of the different leaning postures, the vibration reduction ratios between the experimental measurements and the simulation calculations are very close to each other. The model-predicted vibration reduction effects of the backrest are verified experimentally.

In the experimental mean STHT curves, the resonance frequency of the seat backless (${a}_{0}$) model is close to that of Adam’s experiment^21^ but differs from the simulated values. It is hypothesized that the reason for this is that the parameters quoted in the simulation model originated from Westerners, while the subjects in this paper are Asians. The STHT resonance frequency of Western^21–23^ subjects was around 5 Hz, while that of Asians^24–26^ was around 3 Hz, which is close to the simulation and experimental results in this paper.

In terms of peak reduction effects, the experimental and simulation results for supporting the head (${a}_{1}$) were closest, and the experimental results for supporting the chest back (${a}_{2}$) and supporting the waist back (${a}_{3}$) both differed from the simulation results by less than 5%. The errors may be due to changes in muscle status. Although the experiment tried to select sitting positions that could maintain the muscle state, the backrest would always make people unconsciously relax, which was difficult for the subjects to overcome.

Since the experimental excitation is sinusoidal and the amplitude is constant, this results in a quadratic increase in acceleration with frequency. After 4 Hz, when the backrest touches the head, most of the experimental subjects reported discomfort, mainly because the head is squeezed by the aluminum profile, which indicates that the force capacity of the head is not as good as that of the chest back and waist back. Therefore, when improving the STHT curve, it is also necessary to consider the force capacity of different parts of the human body.

### Effect of backrest contact stiffness and damping on STHT

The support of the chair back can effectively reduce the vibration response of the seated human body. However, when the chair back stiffness is too large in the experiment, the subject’s head will feel uncomfortable due to compression. In this section, the influence law of these two parameters, contact stiffness and damping, on the vibration response of the seated human body within a certain range will be discussed.

The model in full contact with the backrest (${a}_{7}$) is adopted as the object of discussion and analysis. Wan’s seat contact stiffness and damping were scaled once each at 1/3 times the original values as the upper and lower bounds of the interval. The range of stiffness is taken as 37–66 kN/m and the range of damping is taken as 1900–3300 Ns/m. The contact stiffness between the human body and the seat is set to be equal, as is the damping. The change rule of STHT is investigated by simulation calculation.

**Figure 10:**
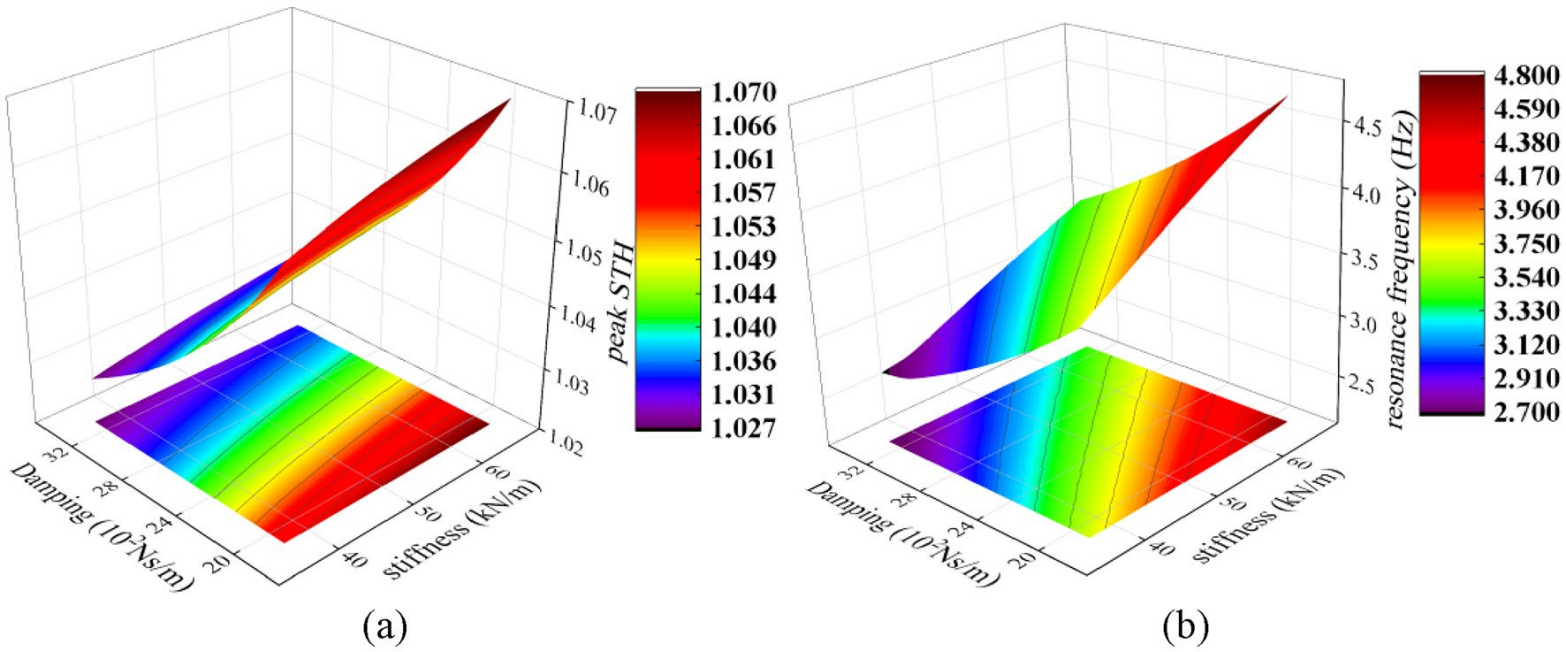
STHT biodynamic response of the full-contact model (${a}_{7}$): (1) peak; (2) resonance frequency.

Based on the range of values of stiffness and damping, the 3D variation in the peak STHT curve and resonance frequency is plotted by simulation, as shown in Fig. 10.

Figure 10 shows that the greater the contact stiffness of the seat material, the smaller the contact damping, and the greater the peak and resonance frequency of the STHT curve. There is a positive correlation between the STHT peak and resonance frequency and stiffness, and a negative correlation with damping.

## Discussion

In engineering, the excitation to which the human body is subjected can be categorized into periodic and nonperiodic excitation. Subject to periodic excitation near the resonant frequency, the smaller the peak of the STHT curve, the better. For nonperiodic excitation, the total energy of the STHT response of the occupant should be as small as possible. The full contact model (${a}_{7}$) is taken for the numerical optimization study under two operating conditions, and the stiffness and damping range of the backrest is referred to in “Effect of backrest contact stiffness and damping on STHT” section.

### Periodic excitation

To solve for the minimum STHT curve peak, first take the set of all STHT peaks in the frequency range of 1 to 20 Hz, and then take the minimum of them.

8 $$\begin{gathered} {\text{min}}\_STHT_{{{\text{max}}}} = {\text{min}}\left\{ {{\text{max}}\left( {STHT\left( {K,C,f} \right)} \right)} \right\} \hfill \\ K = [k_{10} ,k_{20} ,k_{30} ,k_{40} ]^{T} ,\;C = [c_{10} ,c_{20} ,c_{30} ,c_{40} ]^{T} \hfill \\ \left\{ {\begin{array}{*{20}l} {37 {\text{ kN/m}} \le K \le 66{\text{ kN/m}}} \hfill \\ {1900{\text{ Ns/m}} \le C \le 3300 {\text{ Ns/m}}} \hfill \\ {1{\text{ Hz}} \le f \le 20{\text{ Hz}}} \hfill \\ \end{array} } \right.. \hfill \\ \end{gathered}$$

After calculation, when the stiffness is taken as 37 kN/m and damping is 3300 Ns/m, the peak of the STHT curve is minimized to 1.023 and located at 2.31 Hz.

### Nonperiodic excitation

The total energy of the STHT response is as small as possible, i.e., the area under the curve is as small as possible. Parameter identification is performed using the integral equation.

9 $$\min\, F=\int \left|STHT\right|df.$$

$f$ stands for frequency, which is in the range of 1 to 20 Hz.

By calculation, the area under the STHT curve is minimized when the chair back has a stiffness of 37 kN/m and a damping of 1900 Ns/m, with a peak value of 1.065 at 3.60 Hz.

**Figure 11:**
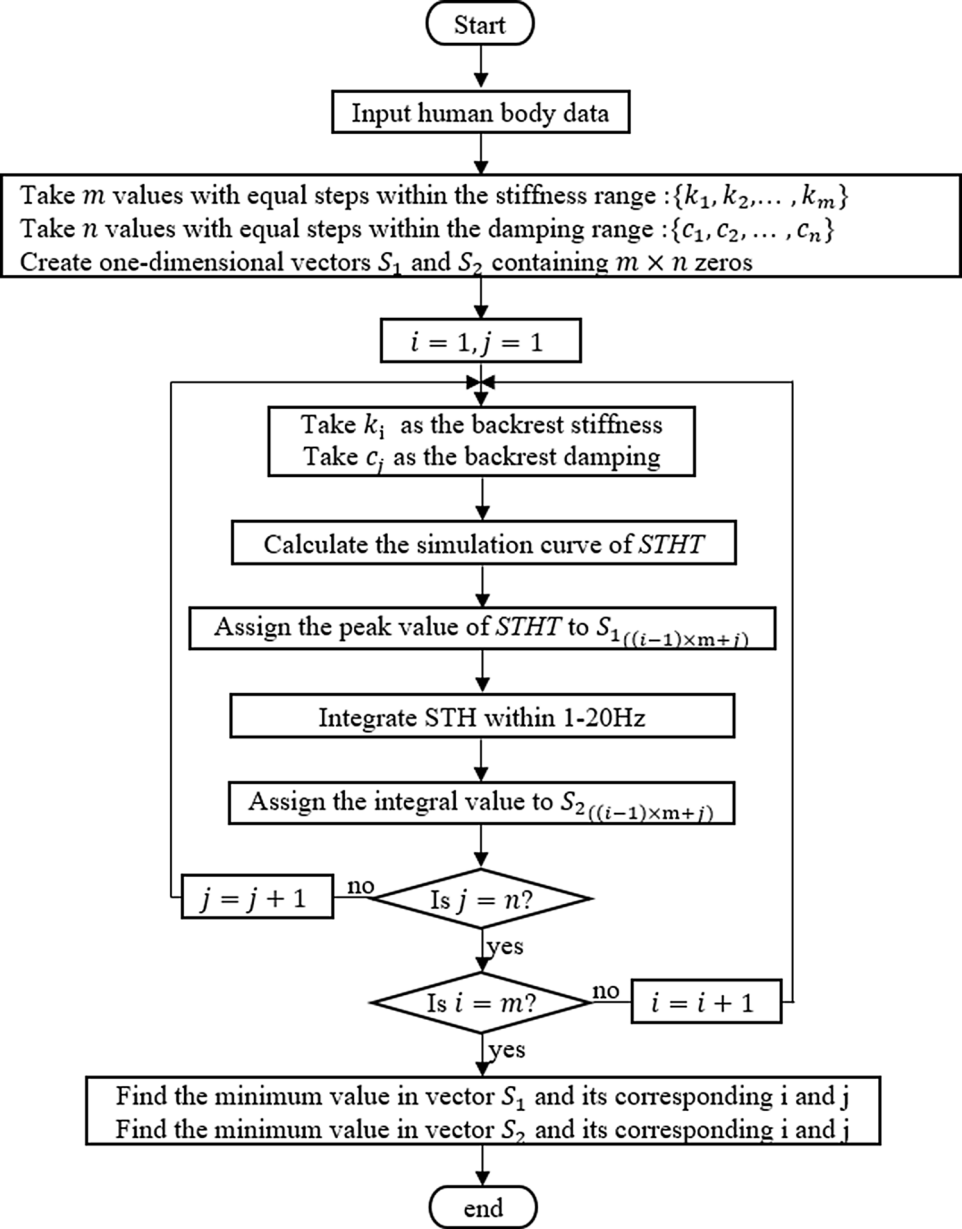
Flowchart for solving the contact stiffness and damping of backrest under periodic and nonperiodic excitation.

Figure 11 shows the solution for the contact stiffness and damping of the seat backrest for two excitation conditions. In summary, the seat back contact stiffness should be as small as possible within the permissible range, and the damping should be as large as possible under periodic excitation and as small as possible under nonperiodic excitation.

## Conclusions

To study the vibration reduction effect of the backrest on the human bod, this paper establishes 4-DOF sitting models characterizing the different contact modes between the backrest and the upper torso, carries out simulation calculations of their vibration response, and conducts experiments on simple sitting postures that easily maintain the muscle state. Taking the full-contact model (${a}_{7}$) as an example, the influence laws of the backrest contact stiffness and damping on the peak value of the seat-to-head transfer rate curve and the resonance frequency are investigated by simulation. The selection methods of backrest contact stiffness and damping under periodic and nonperiodic excitation conditions are proposed.

The main conclusions of this paper are as follows:

1. The experimental curves for the selected postures were close to the peak vibration reduction effect of the simulated curves, demonstrating that the models can be used for STHT prediction in different sitting postures.
2. When the upper torso is supported by the backrest, the peak of the STHT curve will be significantly reduced (P < 0.05). When a single support point is used for the backrest, supporting the head (${a}_{1}$) has more than 20% vibration reduction amplitude and is the most effective, followed by supporting the chest back (${a}_{2}$), and supporting the waist back (${a}_{3}$) is the least effective. These regularities can be used as a reference for seat backrest design.
3. Both the contact stiffness and damping of the chair surface affect on STHT. The peak value and resonance frequency of the STHT curve are positively related to the contact stiffness and negatively related to the contact damping.
4. Under cyclic excitation, use the minimum peak STHT as a criterion for seat stiffness and damping design. Under non-periodic excitation, use the minimum energy of STHT within the excitation frequency as a criterion for design.

## Data Availability

The datasets used during the current study are available from the corresponding author on reasonable request.
